# Antioxidant activity of five Brazilian plants used as traditional medicines and food in Brazil

**DOI:** 10.4103/0973-1296.71789

**Published:** 2010

**Authors:** Allana K. L. Santos, José G. M. Costa, Irwin R. A. Menezes, Isaac F. Cansanção, Karla K. A. Santos, Edinardo F. F. Matias, Henrique D. M. Coutinho

**Affiliations:** *Laboratory of Research on Natural Products, Brazil*; 1 *Laboratory of Pharmacology and Medicinal Chemistry, Brazil*; 2 *Laboratory of Microbiology and Molecular Biology, University of Region of Cariri, Crato (CE), Brazil*; 3 *College of Medicine, Federal University of Ceará, Barbalha (CE), Brazil*

**Keywords:** DPPH, *Eugenia jambolanum*, *Eugenia uniflora*, *Hyptis martiusii*, *Mentha arvensis*, Momordica charantia, *radical scavenging*

## Abstract

**Background:**

This study evaluates the radical-scavenging activity of five plants used as food and medicines in the northeastern region of Brazil.

**Materials and Methods:**

Spectrophotometric analysis of the plants’ ethanol extracts was carried out. The antioxidant activity was determined by the DPPH (2,2-diphenyl-1 picrylhydrazyl) test. The antioxidant capacity was measured using ascorbic acid as a positive control.

**Results:**

All tested plant extracts showed an antioxidant activity, but the highest activity was observed with the extracts of **Momordica charantia** and **Eugenia jambolana**.

**Conclusions:**

Therefore, these species must be studied as a putative source of products for use in the prevention and treatment of diseases in which oxidants or free radicals are implicated.

## INTRODUCTION

All biological systems, with the exception of the anaerobic microorganisms, produce reactive oxygen species (ROS) as a result of energetic metabolism.[1,2] These ROS interfere with some biochemical systems of cells.[3] The most common examples of ROS are the superoxide (O_2_^-·^), hydroxy (OH^·^), peroxy (RO_2_^·^), alkoxy (RO^·^), and hydroperoxy (HO_2_.) radicals.[4,5] Other molecules such as hydrogen peroxide (H_2_O_2_) and peroxynitrate (ONOO) are not free radicals, but they can lead to their production through chemical reactions.[6] Oxidative stress (OS) corresponds to a discrepancy between the production of oxidizing agents and their degradation.[7] Genetic predisposition, environmental factors such as UV radiation, and specific intrinsic properties of cellular groups can enhance the oxidative damage or lower the capacity of cells to degrade these agents.[8,9]

Lipidic peroxidation is the main consequence of oxidative stress. It results in the damage of the membranes due to an increase in the fluidity of the membrane, affecting its integrity and inactivation of the interaction between the receiving membrane and enzyme membrane.[10] Published data indicate that chronic degenerative diseases such as Alzheimer’s and Parkinson’s diseases, atherosclerosis, complications in the diabetes mellitus, precocious aging, and others are related to oxidative stress.[2] Antioxidants represent a group of substances that, when present in ideal concentrations in oxidant substances or foods, inhibit or delay the oxidative processes, being able to be divided in enzymatic, soluble, nutritional, and scavenging metals of transitions.[11]

Because the natural antioxidant mechanism in mammals can be inefficient under some circumstances, a dietary intake of antioxidant compounds is an alternative to combat oxidative stress. A previous study described an inverse relationship between the dietary intake of antioxidants and the incidence of diseases caused by the deficiency of these substances.[12] In recent years, synthetic antioxidants such as butylated hydroxyanisole (BHA) and butylated hydroxytoluene (BHT) have been added to food preparations because they are good free radical scavengers, even though there is some experimental evidence that they induce DNA damage.[13] Therefore, there is an increasing interest in discovering antioxidants derived from natural origins to prevent oxidative stress produced by ROS and reactive nitrogen species (RNS).[14]

The essential oil from leaves of *Hyptis martiusii* was tested against *Bemisia argentifolii* and *Aedes aegypti*. This plant shows antibacterial, antibiotic modifier and phototoxic activity.[15–17] The insecticidal activity of the major component isolated from the essential oil of the leaves, 1,8-cineole, is also reported.[18] The volatile extract of peppermint leaves (*Mentha arvensis* L.) is used as an ingredient in various foods, as an antibacterial agent, as a modulating agent for the antibiotic activity, and as a promoter of gas secretion.[19,20] **Momordica charantia** Linn (bitter gourds) (Cucurbitaceae) is widely cultivated for its medicinal and food uses. The fruits (bitter gourds) of the plants are used in culinary preparations all over the world[21] and they have been previously shown to induce apoptosis in HL60 human leukemia cells along with other biological activities.[22–24] **Eugenia jambolana** L. (Myrtaceae) is native to tropical Asia and was introduced into Brazil. The powder obtained from the seeds has found some popular uses in the treatment of diabetes.[25] The results from the current study warrant further investigation into the potential of *E. jambolana* berries (and their derived food products) to serve as chemotherapeutic agents against breast cancer as well as a putative products to be used in photodynamic therapies.[17,26] The essential oil derived from *Eugenia uniflora* L. (Myrtaceae) has been used in folk medicine to treat digestive disorders, fever, and rheumatism. Infusions prepared from Nangapiry are used in folk medicine as diuretic, antifebrile, and antidiabetic preparations.[27] The essential oil obtained from the leaves of *E. uniflora* exhibits an antimicrobial activity.[28]

From this point of view, the main goal of this research was to study the antioxidant activities of these compounds through the 1,1-diphenyl-2-picrylhydrazyl (DPPH) radical-scavenging method of crude ethanol extracts from five plant species belonging to three botanical families (Myrtaceae, Lamiaceae, and Cucurbitaceae). The plants were collected from the Cariri region in the southern part of Ceará state, in the northeastern region of Brazil, and all of them are used as traditional medicines and foods by the populations of this region. Our efforts are aimed toward the identification of the biological activities of these plants, which have the potential to be a readily available, low-cost alternative to the current therapeutics.

## MATERIALS AND METHODS

### Materials

The ethanol was of analytical grade and supplied by Reagen; dimethyl sulfoxide was purchased from Merck (Darmstadt, Germany); DPPH in the free radical form was obtained from Aldrich Chemical (Milwaukee, WI, USA).

### Plant material and extract preparations

Leaves of each species were collected in the county of Crato, Ceará State, Brazil. The plant material was identified and a voucher specimen was deposited with the respective numbers at the Herbarium ℌDárdano de Andrade Lima” of Universidade Regional do Cariri – URCA [Table 1]. A quantity of 200 g of aerial parts was dried at room temperature and powdered. The powdered material was extracted by maceration using 1 L of 95% ethanol as a solvent at room temperature, and the homogenate was allowed to stand for 72 h at room temperature. The extracts were then filtered and concentrated under vacuum in a rotary evaporator (model Q-344B – Quimis, Brazil) and ultrathermal bath (model Q-214M2 – Quimis, Brazil). Each 200-g quantity of aerial parts yielded 5–6 g of the extract [Table 1].

**Table 1:** Botanical families, species, and voucher number of the plants used in this study

Family	Species	Abbreviation	Number
Lamiaceae	Hyptis martiusii	EEHM	#464
Lamiaceae	Mentha arvensis	EEMA	#2886
Cucurbitaceae	**Momordica charantia**	EEMC	#703
Myrtaceae	Eugenia jambolanum	EEEJ	#3107
Myrtaceae	Eugenia uniflora	EEEU	#3106

**Table 2:** CE_50_ values in the DPPH scavenging activity

Extracts and substance	CE_50_ (μg/mL)^a^
Ascorbic acid (standard	0.82 ± 0.03
EEMA	63.38 ± 2.75
EEEU	179.77 ± 2.04
EEEJ	25.40 ± 0.113
EEMC	9.64 ± 0.045
EEHM	134 ± 7.6

a The results are expressed in CE_50_ ± error standard of the average

### Measurement of the DPPH radical-scavenging activity

**Figure 1:**
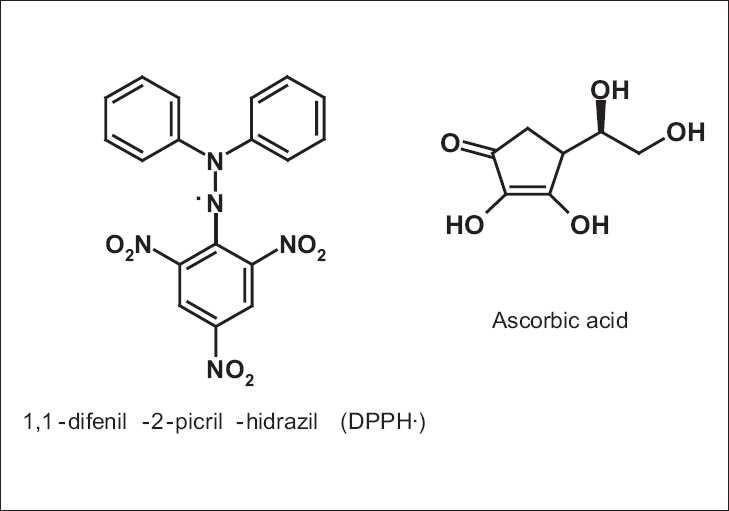
Structures of DPPH and ascorbic acid

The measurement of the radical-scavenging activity was accomplished using the adaptation of the methodology described by Silva *et al*.[29] The stock storage of the extracts was prepared at a concentration of 1.0 mg/mL. Three milliliters of the DPPH solution (23.6μg/mL in EtOH) was placed in a 5-mL volumetric balloons (10 balloons) and the appropriate amounts (obtained through the preliminary selection) of these extracts were transferred to these balloons, supplying final concentrations that varied from of 5 to 500 μg/mL. Each concentration was tested in triplicate. After 30 min of agitation in an ultrasound device, the amount of DPPH radicals was measured by monitoring absorption at 517 nm in a UV-visible spectrophotometer. The percentage of the scavenging activity (%) was calculated using the following equation:

$$\%\mathrm{AS}\;=\;100\;\times \;(A_{\mathrm{control}}\;-\;A_{\mathrm{sample}})/A_{\mathrm{control}}$$

where *A*_control_ is the absorbance of the control (a solution that contains only radical DPPH and EtOH) and *A*_sample_ is the absorbance of the radical in the presence of the extracts or the ascorbic acid and BHT standards. The scavenging efficiency of the radicals was established by a linear regression in the reliable break of 95% (*P*<0.05) obtained by the statistical program GraphPad Prism 4.0. The results are expressed as CE_50_ ± EPM, a unit which represents the concentration of the sample necessary to obtain half of the maximum scavenging activity. The extracts are considered active when CE_50_ <500 μg/mL.[30] Ascorbic acid was used as the positive control [Figure 1].

## RESULTS AND DISCUSSION

DPPH reduction was evaluated by decolorization of the radical solution (absorbance in the range of 515–528 nm) in the presence of the plant extract.[31] Because the DPPH radical can accommodate many samples in a short period of time and is sensitive enough to detect active molecules at low concentrations, it has been extensively used to screen the antiradical activities of vegetal extracts, juices, and fruits.[32]

The presence of previously isolated phenolic metabolites such as flavonoids, cathechins, tannins, triterpenoids, oxygenated sesquiterpenes and oxygenated monoterpenes, phenolic acids, and alkaloids justify the observed effects that are presented below. DPPH exists as a purple, free organic radical that can accept an electron or as a visually distinct free radical species that is yellow in color. To become a stable diamagnetic compound, because of its odd electron, the ethanol solution shows a strong absorption band in the range of 515–528 nm. As DPPH reacts with suitable reducing agents, this electron becomes paired off and the solution loses color stoichiometrically with the number of electrons taken up. Such reactivity has been widely used to test the ability of several compounds to act as free radical scavengers and to probe the antioxidant activity of plant extracts.[31]

This study determined the antioxidant activity of five species [Table 1] of the families Lamiaceae, Cucurbitaceae, and Myrtaceae. The results indicate that the ethanol extract of all the plants demonstrated antioxidant activity, but the extract of *M. charantia* showed the highest activity among the species studied with a CE_50_ value (the concentration necessary to obtain half of the maximum scavenging activity) of 9.64 ± 0.045 μg/mL. This highest activity was followed by that of *E. jambolana* (25.40 ± 0.113 μg/mL) [Table 2].

The results from previous phytochemical research indicate that all plant extracts contain phenolic compounds such as tannins and flavonoids, substances with an antioxidant activity.[18,25,27,33–36] These polyphenolic compounds are widely distributed in the plant kingdom and they have been shown to possess strong antioxidant properties.[37–40] The antioxidant activities of the extracts of *H. martiusii* and *M. arvensis* are reported here for the first time.

The results of this study suggest that *H. martiusii, M. arvensis, M. charantia, E. jambolana,* and *E. uniflora* are natural sources of antioxidants with the potential for use in pharmaceutical applications.

## CONCLUSION

This study provides evidence that *H. martiusii, M. arvensis, M. charantia, E. Jambolanum*, and *E. uniflora* have antioxidant properties, as confirmed through the DPPH method. Therefore, these species may have great relevance in the prevention and treatment of diseases in which oxidants or free radicals are implicated. In addition, these plants might represent good candidates for further phytochemical and chromatographic studies to isolate and fully characterize the compounds related to this *in vivo* biological activity.
